# Effects of SCUBA bubbles on counts of roving piscivores in a large remote marine protected area

**DOI:** 10.1371/journal.pone.0226370

**Published:** 2019-12-18

**Authors:** Keolohilani H. Lopes, Ivor D. Williams, Randall K. Kosaki, Andrew E. Gray, Jason C. Leonard

**Affiliations:** 1 Papahānaumokuākea Marine National Monument, National Oceanic and Atmospheric Administration, Honolulu, Hawai‘i, United States of America; 2 Joint Institute for Marine and Atmospheric Research, University of Hawai‘i at Mānoa, Honolulu, Hawai‘i, United States of America; 3 Ecosystem Sciences Division, Pacific Islands Fisheries Science Center, National Oceanic and Atmospheric Administration, Honolulu, Hawai‘i, United States of America; Australian Bureau of Agricultural and Resource Economics and Sciences, AUSTRALIA

## Abstract

This study examined the effects of SCUBA bubbles on fish counts in underwater visual surveys conducted in the Papahānaumokuākea Marine National Monument (PMNM). Specifically, paired fish surveys were conducted at each survey site, utilizing two different gear types: open-circuit SCUBA (OC) and closed-circuit rebreather (CCR). Bubble exhaust released from the OC equipment is a potential source of bias for *in-situ* fish observations, as the associated audio and visual disturbances could either attract or repel fishes depending on whether their behavior is more driven by curiosity or caution. The study area, is a large (~1.5 million km^2^) and extremely remote marine protected area in which the response of coral reef fishes to divers represent natural behavior of naive fishes with little or no previous contact with humans. Historically, surveys conducted on OC in this area have shown an abundance of large roving piscivores and this study set out to determine the extant, if any, the audible and visual disturbances of OC bubbles have. The species typically seen in these prior surveys were *Caranx ignobilis*, *Caranx melampygus*, *Aprion virescens*, and a couple of species of sharks. We found differences in counts for some roving piscivores, including significantly more jacks observed on OC than CCR (*Caranx ignobilis* 57% more, and *Caranx melampygus* 113% more). Instance of first encounter, i.e. the time when a fish was first observed during a survey, also varied for some species. Higher numbers of *Aprion virescens* (p = 0.04), and *C*. *melampygus* (p = <0.001) were observed in the first 5-minutes of counts by divers on OC (i.e. when they were using breathing apparatus that produced noises that could be heard over long distances). Although not the focus of the study, we also assessed differences between OC and CCR counts for other groups of fishes. Estimated abundance of benthic damselfish was higher on OC than CCR, and counts of butterflyfish were lower on OC; but there were no significant differences for the other groups considered. This is an important control study that documents the natural responses of coral reef fishes to SCUBA bubbles generated by *in-situ* surveys.

## Introduction

The scientific inquiry of marine life poses unique challenges. Potential sources of bias associated with *in situ* visual fish surveys are numerous, including diver experience level [1,2], survey method [3], differences in fishing pressure among locations and human populations [4,5] Survey divers typically use open circuit SCUBA (OC) equipment that releases exhaust bubbles, and introduce visual disturbances and audible SCUBA exhaust, that has been measured at 90 dB [6] and can be heard by fishes as far as 200 m away [7]. This noise, in addition to the visual disturbances of the exhaust bubbles, introduce a constant and potential bias present from the first modern era *in-situ* fish survey [8]. Conversely, the closed-circuit rebreather (CCR) emits virtually no bubbles, effectively eliminating these audible and visual disturbances. Despite the bias that OC exhaust may introduce in fish surveys, it is widely used due to the durability, ease of use, and the relatively low cost to operate. However, with the increased accessibility of electronic closed-circuit rebreathers (CCR), the elimination of OC exhaust is now safer, more affordable, and is becoming more common in research, providing a means to eliminate one source of potential bias to fish surveys. With the increased use of CCRs in fish research, it is imperative to understand how the lack of OC exhaust affects observations for different taxa and in different locations.

Previous research on the effects of SCUBA exhaust on fish counts have had a range of results varying from no difference, to very large differences between OC and CCR. These previous studies utilized different survey methods, in different regions of the Pacific Ocean, and at locations with varying levels of fishing pressure. Fishing pressure tends to increase the flight response of fishes, presumably due to the perceived threat from human divers based on earlier life experiences [4, 9]. This heightened flight response may repel and reduce the number of fishes counted in these surveys. Recent studies by Gray et al. [10] and Lindfield et al. [5] both concluded that the degree of fishing pressure affects the magnitude of differences in counts between the gear types, but with large differences between those studies in the size of that impact. Lindfield et al. [5] reports that counts of fish groups targeted by fishers were 200–300% higher on CCR in fished areas, whereas the differences observed by Gray et al. [10] were more modest at around one third fewer fish observed in the most fished areas. In contrast, Cole et al. [11] found no differences between fish surveys utilizing a semi-closed circuit rebreather and OC SCUBA.

In this study, we assess impacts of breathing apparatus on surveys in the PMNM which is free from any fishing pressure that could alter behavior, such as freediving or spearfishing. Specifically, Papahānaumokuākea Marine National Monument (PMNM) is one of the largest marine protected areas in the world with over 1.5 million km^2^ of fully protected waters and islands in which no fishing of any kind is allowed [12]. Additionally, this area requires permits for access and there is very little to no boat activities besides annual research expeditions. Protected since 2000 via Presidential Executive Orders 13178 and 13196, PMNM has been free from any fishing pressure for over 19 years, but throughout most of its range, coral reef fishes were likely very lightly if at all fished even before then, due to isolation–as the islands and atolls are unpopulated, other than by scientists or resource managers, and are several hundreds to thousands of kilometers away from the nearest human population centers. Its geography and restricted access to this large marine area also eliminates nearly all contact from any type of human activity. Thus, the waters of PMNM, encompassing the Northwestern Hawaiian Islands, are populated with fishes that have likely never encountered spearfishers of any kind and thus have had no reason to associate divers or boats with the threat of being targeted; and, instead, the presence of divers is likely to be a novel stimulus. This creates an ideal study location to observe the instinctual reactions of fishes, without any learned avoidance behavior. Prior experience and studies conducted in this region shows a dominance of roving piscivore species and an attraction to divers [13]. It has also been documented that these apex predators display klepto-parasitic feeding behaviors as documented on video cameras mounted on the Hawaiian Monk Seal [14]. These documented behavioral traits by these roving piscivores have important ramifications for how scientists and managers think about what the species composition of a natural ecosystem should be like and to better interpret their fish data. Our investigation was focused on determining what effects SCUBA exhaust had on the counts of these roving piscivores. Our secondary interest was to more broadly asses the differences between OC and CCR for other groups of fishes. The unique geographic location and protection status allowed us to examine the differences between surveys conducted using the two gear types, OC and CCR, and attribute any differences solely due to natural fish responses associated with OC bubbles. The metrics used to determine the differences will be, primarily, fish abundance due to the relatively large body mass of most apex predators in this region, then biomass, and also species richness.

## Materials and methods

### Study location & surveys

**Fig 1:**
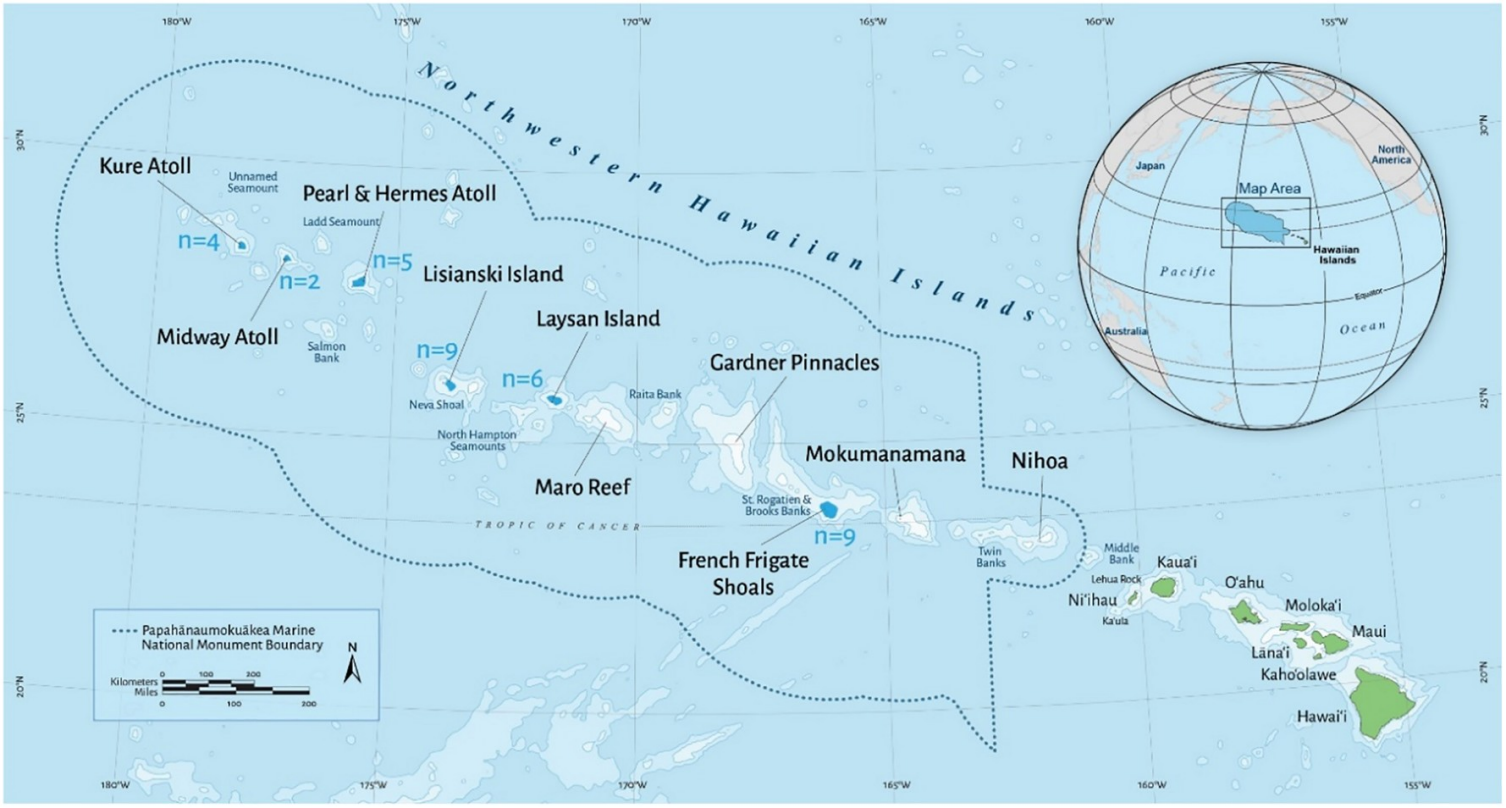
Map of survey sites. Papahānaumokuākea Marine National Monument is located within the dotted lines and is ~ 1.5 million km^2^. In total, 35 paired surveys were conducted at 6 atolls—n values are the number of surveys by atoll.

PMNM spans ~2,500 kilometers in the central Pacific in a northwestern orientation from the main Hawaiian Islands [12] (Fig 1). This archipelago is comprised of 10 major coral islands, several banks and submerged pinnacles. Surveys for this study were conducted as part of a larger research cruise that visited six islands and atolls between September 9^th^ and September 28^th^, 2017. In total, surveys were conducted at 35 sites: Lalo (n = 9, French Frigate Shoals), Kamole (n = 6, Laysan), Kapou (n = 9, Lisianski), Manawai (n = 5, Pearl and Hermes Atoll), Kuaihelani (n = 2, Midway Atoll), and Hōlanikū (n = 4, Kure Atoll). At each atoll, survey locations were randomly selected in hard-bottom habitat between 9 and 30 m deep (Table in S1 Table). The majority of these dive sites were coral reef ecosystems with high structural complexity that is typical for this region. There were a few (~ 3) survey locations that could be classified as having low structural complexity.

### Dive equipment

The two dive modes used for this study were conventional OC and CCR. The CCR units used were the Inspiration from Ambient Pressure Diving Ltd (Cornwall, UK) and the Poseidon Se7en (Poseidon Diving Systems AP). Both of these CCR units are completely closed loop systems that are exhaust-free at constant depth, therefore eliminating the auditory and visual disturbances associated with exhaust bubbles [15].

### Survey method

For this study, fish surveys were conducted using a stationary point count (SPC) that is used by the National Oceanic and Atmospheric Administration (NOAA), Pacific Reef Assessment and Monitoring Program (RAMP) which spans the entire Pacific [16]. In brief, the method involves two divers who descend at a randomly selected dive site, and roll out a 30 m transect line along a depth contour. Each diver collects visual observations of fishes within an imaginary 15 m diameter cylinder, with one diver centered at 7.5 m and the second diver centered on the 22.5m mark on that transect. After establishing their positions, the divers signal each other and begin the survey. During the first five minutes of the survey the divers generate a list of all fish species observed within their cylinder. After the first 5 minutes has elapsed the divers enumerate each species starting from the beginning of the list. The quantity and size of each species is recorded during a “snapshot” in time (attempting to generate near-instantaneous counts per species). While this quantification process is occurring, if a new species enters the cylinder, the quantity and size are noted in addition to the time period it entered the cylinder (5–10 min, or > 10 min). The survey is complete when the initial species list has been exhausted and all the species have been quantified, typically around 30 minutes after starting the survey.

In order to replicate the methodology used by RAMP, the divers were deployed in the exact same manner. The divers prepared to enter the water, the boat would then go to the GPS location, then deploy both divers. This would more closely simulate the RAMP experimental design and increase the safety of the divers. The methodology of this study was chosen in order to replicate the approach taken by Gray et al. [10]. Each transect was surveyed with both gear types, OC and CCR. After the completion of the first survey using one gear type, the transect line was left in place. The same divers then conducted a second survey with the other gear type, leaving ~30-minutes between surveys. The starting gear type was randomly selected prior to each day’s activities, then divers alternated the gear types for the remainder of the day. There were typically 3 sites surveyed per day (range 2 to 4). To reduce observer variability, the same two, highly experienced fish surveyors conducted the majority of the survey dives, with the exception being two days during which a third diver with an equivalent level of training was used as a substitute. From these observations, biomass is calculated by utilizing the fish species length to average weight ratio using as calculated by Kulbicki 2005 and Froese 2015 [17, 18].

### Statistical analysis

#### Data pooling

The fish data is organized into groups, first by our primary group of interest, the roving piscivores and the individual fish species which compose the group of Jacks, *Caranx melampygus*, *Caranx ignobilis*, *Aprion virescens*. Additionally, sharks were analyzed both as a group as well as the two individual dominate shark species, *Carcharhinus galapagensis*, and *Trianodon obsesus*. Other groups of fishes were primarily chosen by family and those frequently used by the scientific community. These groups include Jacks, Benthic Damselfish/Angelfish, Butterflyfish, Goatfish, Hawkfish, Midwater Damselfish, Parrotfish, Surgeonfish, Sharks, Triggerfish, Targeted wrasse, and Non-targeted wrasse. The two different wrasse categories, targeted and non-targeted, were separated in order to capture natural behavior trends between the them so other researchers could use this data to assess the natural behavior of the wrasse species that are historically fished in the Main Hawaiian Islands.

#### Relative abundance by gear type

For the purposes of this study, fish abundance was used as the primary response variable, but to add comparability with other studies, results were also generated for biomass of groups and species of interest. Total species richness–i.e. number of species recorded per survey—was also calculated. Abundance was calculated by dividing the total number of individuals observed on each transect by the total area of the two surveyed cylinders (~354 m^2^) and results are shown as number of fish per hectare (#/Ha). Confidence intervals of abundance or biomass per breathing apparatus and confidence intervals of difference between breathing apparatus were calculated using a bootstrapping approach in R 3.4.2 [19] using the *boot* package [20]. Specifically following the methods of Gray et al. [10], for each response variable of interest (e.g. abundance of a particular taxa) we used bootstrapping with 10,000 iterations to generate quantile ranges of the mean of that variable, and of the difference between the OC and CCR counts for that variable. Differences between methods were converted to ratios of difference–specifically the OC:CCR abundance ratio (AR) is a measure of the difference between OC and CCR counts as a proportion of the CCR count, and similarly for biomass rations (BR). The quantile ranges of the difference between breathing apparatus are equivalent to confidence intervals; and differences in counts between breathing apparatus were considered significant when the 95% confidence interval of the AR or BR did not overlap 1 (i.e. the ratio was therefore either significantly higher or lower than 1) with 95% confidence. Bootstrapping is particularly suitable for this analysis as: (1) counts are highly non-normal for the majority of taxa and groupings of interest; and (2) because we were primarily interested in the extent to which different breathing apparatus either inflated or diminished the counts–i.e. the proportional change–and while it may have been possible to transform the data and perform parametric test, transformation of data followed by back-transformation of results modifies the estimation of effect size. Probably for that reason, approaches similar to ours have been widely used to detect effect sizes in coral reef studies [21,22]. Graphical representations of these results were created with the ggplot2 [23] package for R [19].

#### Distribution of instance of first encounter

In addition to the analysis for differences in fish abundance between gear types, the distribution of counts within different periods of the survey were compared for a number of common taxa. As described above, data gathered during surveys include the time period in which a species was first observed: either “Five” if it arrived during the initial 5 minutes of the survey, “Five to Ten” if it arrived between 5 and 10 minutes, or “Ten” is if a species was first observed between 10 and 30 minutes. For five species of interest (*A*. *virescens*, *C*. *galapagensis*, *T*. *obesus*, *C*. *ignobilis*, and *C*. *melampygus)*, the distribution of counts across those time periods for OC and CCR were compared using a Chi-squared test. These are all highly mobile predatory species, and were selected due to our expectation that they had greatest potential for behavioral differences in response (either attraction or repulsion) to divers on OC and CCR.

## Results

### Ratio of differences

**Fig 2:**
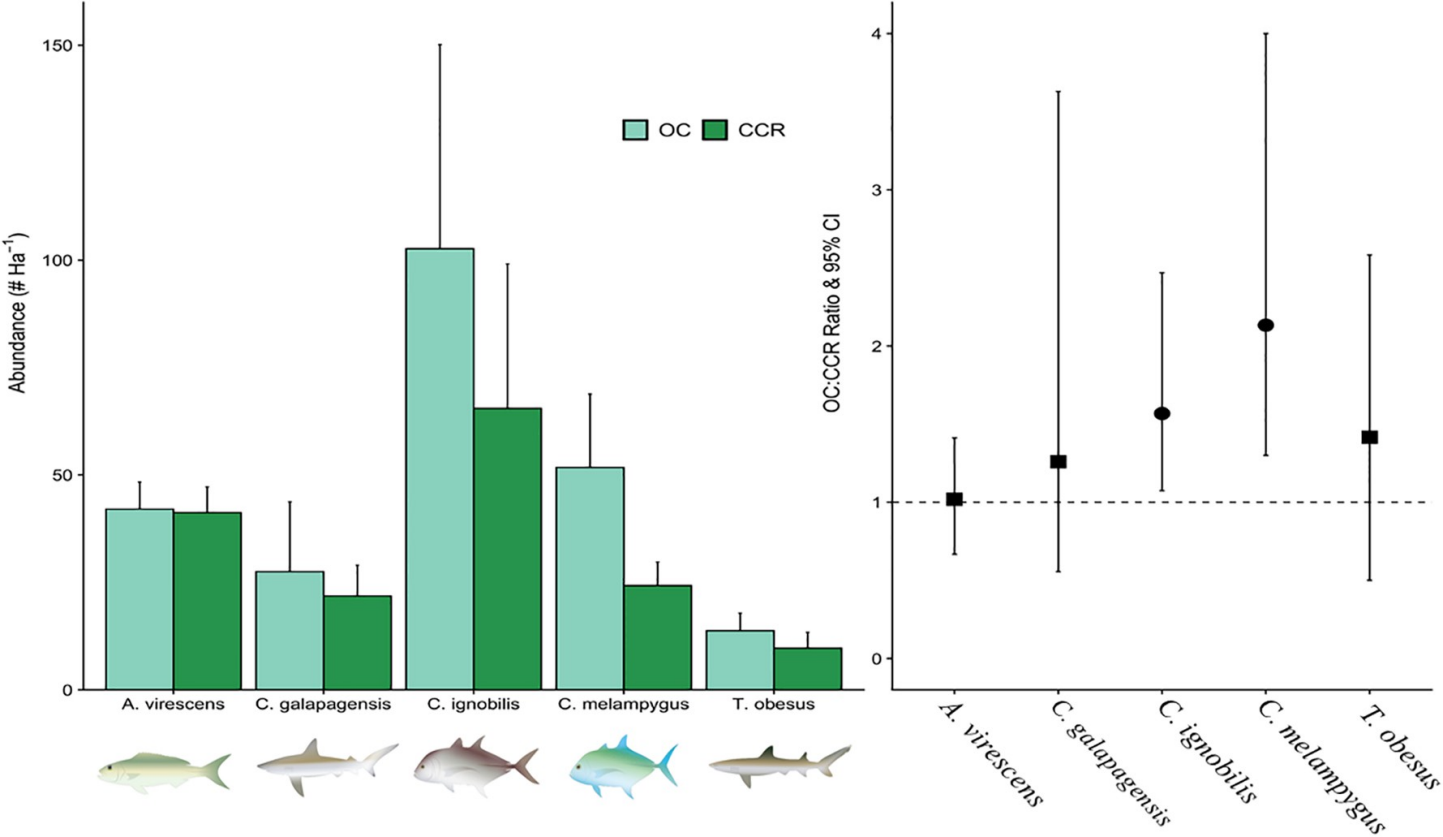
Abundance of fish species between gear types. Densities per species and relative abundance on open circuit SCUBA (OC) and closed-circuit rebreather (CCR). The left hand figure shows mean and standard error of abundance per species. The right hand figure represent the mean and 95% confidence intervals of the OC:CCR ratio (i.e. abundance in OC surveys relative to abundance in CCR surveys). Mean values above 100% indicate higher counts on OC. Ratios are considered significant when the 95% confidence intervals of the ratio do not overlap the 100% line.

We found no significant differences (95% confidence interval (CI) overlaps 1) between the two gear types for total fish biomass (OC:CCR BR:1.29, 95%CI:0.85, 1.57, Table in S2 Table), species richness (OC:CCR:0.97, 95%CI:0.93, 1.01), and abundance (OC:CCR AR:0.92, 95%CI: 0.66, 1.06). The majority of the fish species and groups also showed no significant difference between the breathing gear types with a few exceptions (Table in S3 Table)(Figure in S1 Fig). Fish groupings with higher abundance on OC were the benthic damselfish/angelfish (AR: 1.37, 95%CI: 1.06, 1.95) at ~40% more, the primary driver for these differences was a single species *Chromis hanui*, it accounted for 1529 of the total 2138 individual fishes counted on OC (Table 1). The trevally jacks (AR:1.54, 95%CI: 1.06, 2.43, Table 1, Table in S2 Table) were observed at ~50% more individuals while the *C*. *ignobilis* mean counts were ~57% higher on OC than CCR (AR: 1.57, 95%CI: 1.07, 2.47), and *C*. *melampygus* counts were about double (AR:2.13, 95%CI: 1.30, 4.00, Fig 2). Shark counts tended to be higher on OC, however these differences were not statistically significant (AR:1.32, 95% CI: 0.78, 2.77, Table 1). Conversely, significantly fewer butterflyfishes (AR: 0.79, 95% CI:0.40, 0.95) were counted on OC (Table 1 and Table in S2 Table). Unlike the benthic damsel/angelfish group where just one species was the primary driver of tendency, the butterflyfish group were basically uniform in the tendency to have increased numbers on the CCR. In the case of the *Seriola dumerili* (trevally jacks group), the raw data indicates a higher number of individuals observed on CCR, however, all of these fish were counted on only 3 of the 35 total transects which resulted in an extremely large CI, which renders this species level observations inconsequential (Table 1).

**Table 1:** Fish counts of individuals per group at the species level. All of these groups except the sharks showed significant differences between gear types. The groups of benthic damselfish/angelfish, butterflyfish, jacks, and sharks are listed with total number of fish counted for the 35 surveys conducted on each gear type.

Group	Species	OC	CCR
Benthic Damsel/Angelfish	*Centropyge fisheri*	2	5
	*Centropyge loricula*	1	1
	*Centropyge potteri*	311	326
	*Chromis hanui*	1529	952
	*Plectroglyphidodon johnstonianus*	167	130
	*Stegastes fasciolatus*	128	147
Benthic Damsel Total		2138	1561
Butterflyfish	*Chaetodon auriga*	15	31
	*Chaetodon citrinellus*	1	0
	*Chaetodon fremblii*	48	57
	*Chaetodon kleinii*	15	20
	*Chaetodon lunulatus*	7	12
	*Chaetodon miliaris*	169	220
	*Chaetodon multicinctus*	63	91
	*Chaetodon ornatissimus*	15	23
	*Chaetodon quadrimaculatus*	1	3
	*Chaetodon trifascialis*	60	38
	*Chaetodon unimaculatus*	4	11
	*Forcipiger flavissimus*	23	30
	*Forcipiger longirostris*	4	3
Butterflyfish Total		425	539
Trevally Jacks	*Carangoides ferdau*	1	0
	*Carangoides orthogrammus*	8	5
	*Caranx ignobilis*	127	81
	*Caranx melampygus*	64	30
	*Caranx sp*	0	1
	*Elagatis bipinnulata*	3	0
	*Pseudocaranx dentex*	1	0
	*Seriola dumerili*	4	18
Trevally Jacks Total		208	135
Sharks	*Carcharhinus amblyrhynchos*	2	2
	*Carcharhinus galapagensis*	34	27
	*Carcharhinus limbatus*	1	0
	*Triaenodon obesus*	17	12
Sharks Total		54	41

### Distribution of the instance of first encounter

**Fig 3:**
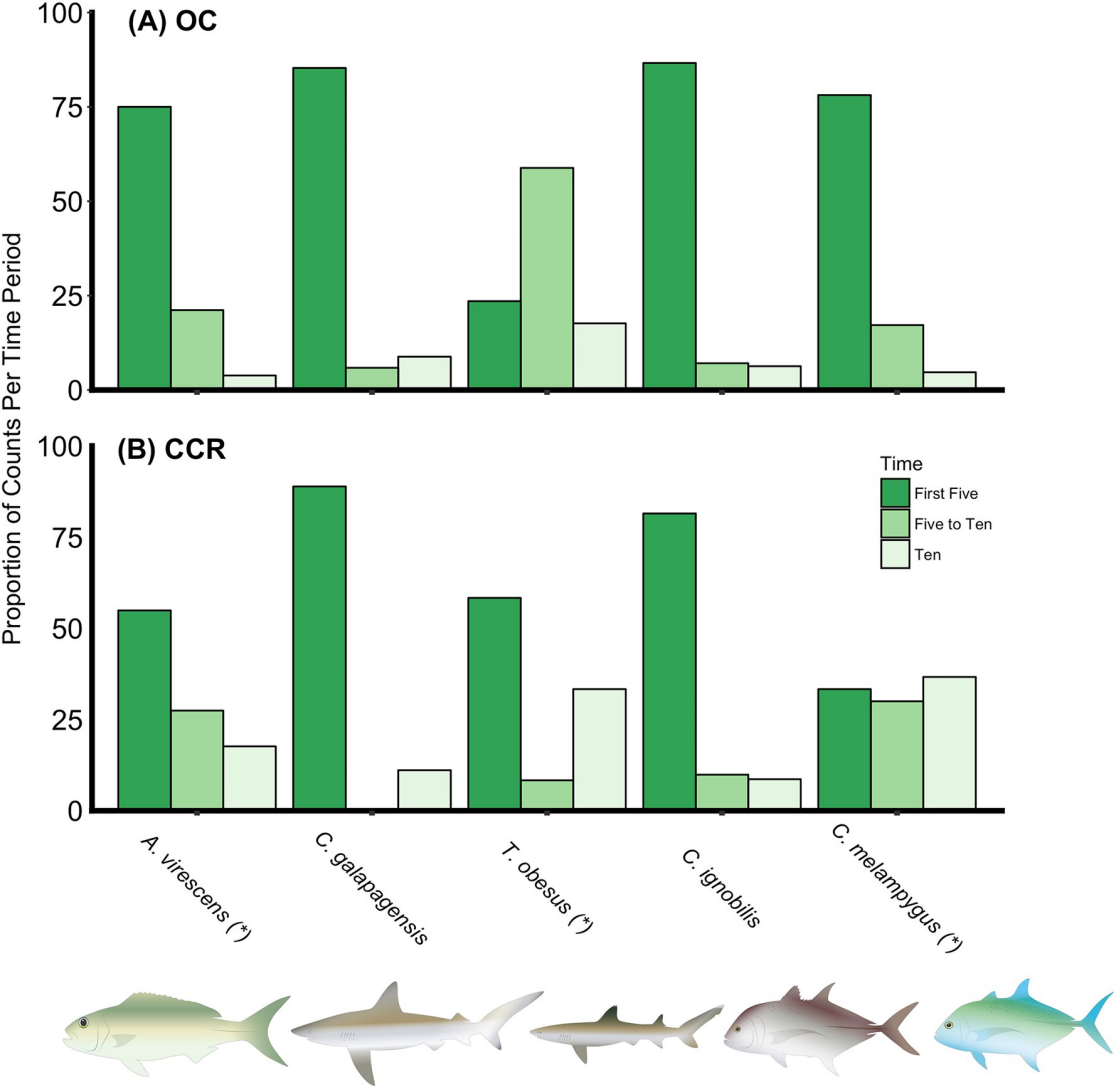
Distribution graph of the instance of first encounter. Survey periods in which roving piscivores were first recorded during open circuit (OC) and closed-circuit rebreather (CCR) surveys. Proportion of counts per time period are shown for 5 species of interest. Different colors represent the 3 time periods: “First Five” represents surveys in which the species was first recorded during the initial five minutes, “Five to Ten” for the second five-minute interval (5 to 10 minutes into survey), and “Ten” for the interval 10 to 30 minutes into the survey. Significant differences in distribution of first encounters between breathing apparatus are denoted by an asterisk (*) after the species name.

*A*. *virescens*, *T*. *obesus*, and *C*. *melampygus* had significantly different distributions of time of first encounter between OC and CCR (p-value = 0.04, 0.02, and <0.001), while *C*. *galapagensis*, and *C*. *ignobilis* both showed similar arrival time distributions (p-value = 0.43 and 0.61, Fig 3 and Table in S4 Table). For all OC counts of all species other than *T*. *obesus*, a large majority (~75% or more) of the first observations during surveys were within the first five minutes of the surveys (Fig 3). On CCR, first observations *C*. *melampygus* were pretty evenly distributed among the 3 time periods (Fig 3)–i.e. tended to be later than on OC. For *A*. *virescens*, there was also a tendency, although less distinct than for *C*. *melampygus*, for first encounter to be a bit later on CCR than OC. In contrast, for *T*. *obesus*, first encounter was more frequently early on CCR (Fig 3).

## Discussion

Previous studies on the effects of SCUBA exhaust on fish surveys have shown little or no difference between counts on OC and CCR in areas of very low fishing pressure, such as small marine protected areas [5,10,11]. Here, we examined this effect in one of the world’s largest marine protected areas, that has not only been fully protected for just over 19 years, but which is also highly remote–being several hundred kilometers from the nearest human population center. Due to its degree of protection and remoteness, coral reef fishes encountered by divers in this study have likely never experienced any fishing pressure and in many cases will not have previously encountered SCUBA divers. The sheer magnitude of the PMNM in combination with its isolation means that resident fishes previous experience of SCUBA divers are fundamentally different to fishes residing in small protected areas. We assume therefore that coral reef fishes within the PMNM have not learned to associate diver presence as a threat, and thus any responses to diver presence are due to their natural curiosity or caution. The results found in this study are consistent with the predictions of the previous studies in that little to no difference in the overall fish biomass or abundance was found between gear types in non-fished areas.

Past research experience on the klepto-parasitic behavior [14] displayed by some roving piscivores led us to believe that there would be significant differences in the numbers of these species between OC and the stealthier, bubble free, CCR. The PMNM is unequalled in the world with regards to its sheer size and its extremely limited human contact resulting in a magnification of the previous findings concerning acquired behavior of reef fish responses to divers [4]. Due to the tendency of MPA’s to be less successful in increasing fish biomass in areas of high human impact [24] it can be inferred that some behavioral traits are causing these observed trends. For our site with an extremely low level of human contact we expect these fishes to have unadulterated behaviors.

What we assume to be the natural curiosity of roving piscivores to an unusual stimulus was manifested in higher counts of *C*. *ignobilis* and *C*. *melampygus* on OC (Table 1, Table in S3 Table). These two species could be initially attracted from the far-ranging sound of the scuba bubbles then hone in to the visual disturbances, as the OC survey observed more individuals for these two jack species and saw them sooner in the counts. The large majority arrived within the “First Five” minute time period when OC was used, whereas CCR seemed to have the opposite effect on several species’ distributions (Fig 3). This was seen in the distribution of *C*. *melampygus* and *A*. *virescens*. The number of fish observed for each of these species were identical between gear types but the instance of first encounter differed significantly. This further substantiates the attraction to the novelty of the OC bubbles by some roving piscivore species. Conversely, the whitetip reef shark (*T*. *obesus*) responded differently than the other predators in that their time of first arrival was later on OC. Notably, this species of shark tends to be much less wide ranging than common jacks and other sharks observed and often rests immobile on the benthos. Thus, it may be less likely to travel longer distances in response to novel sounds (e.g. from OC). For all species, the impacts of OC or CCR gear types on counts likely reflects a mix of potential response to long-distance stimuli (presumably primarily sound) and inherent caution or curiosity about approaching divers closely enough to be recorded in surveys (i.e. within 7.5m).

Learned avoidance due to a perceived threat associated with divers is often assumed to be the primary driver of differences between OC surveys and CCR [9,10,11]. Our study suggests that it is not always the case. For example, we found that divers recorded significantly fewer butterflyfishes on OC than CCR in this isolated, unfished and near pristine ecosystem, which could be interpreted by these species having a natural repulsion for noise or the visual disturbances caused by OC bubbles Thus, even though it may be appropriate to use non-fished butterflyfish as a quasi-control group in some cases [5], it’s important to recognize that counts of unfished taxa can vary between OC and CCR, which likely reflect differences in species composition, natural reaction to audio and visual disturbances, as well as prior exposure to divers.

Another unfished group, benthic damsel/angelfish, showed significantly more fish on the OC surveys. This suggests that this group of fishes were attracted to divers on OC SCUBA. The different responses to the gear types between the butterflyfish and the damsel/angelfish groups show the importance for researches to carefully consider the natural behaviors of each species. Taking a closer look at our grouping results, only one species, *Chromis hanui*, appeared to be driving the differences between the gear types. The rest of the species in that group all show nearly identical numbers between the gear types, further illustrating the need to consider the likelihood of non-trivial differences among apparently similar species, and to be careful about making broad conclusions on fish populations on SCUBA surveys.

Some results from this and other studies suggests that some taxa are relatively indifferent to the presence of divers and the breathing gear, irrespective of fishing pressure. For example, Gray et al. [10] found no differences at 3 locations along a gradient of fishing pressure in the MHI for some groups, including goatfishes. Fishes in that family were also observed in similar proportions for OC and CCR in our study, within both the PMNM and the MHI–i.e. in 35 PMNM surveys 174 goatfishes were recorded on OC and 169 on CCR (Table in S3 Table); similarly, in 66 MHI surveys, 383 were recorded on OC and 375 on CCR [10]. For species that do react differently to divers on OC and CCR, it remains difficult to determine the extent to which those differences are driven by either the audible or visual disturbance associated with OC bubbles. Future studies could usefully attempt to distinguish between those two stimuli, but a more fundamental consideration is that sounds and noise associated with bubble production are of course far from the only disturbances associated with divers conducting a survey–even silent divers are a large and relatively conspicuous presence. Also, use of motorized small boats to move between survey sites, as is common, is another important factor that can likely alter fish behavior and mean that survey counts are not truly representative of natural abundance. Collectively therefore it is important that researchers and managers utilizing survey data clearly recognize the limitations of the data available to them, including generally treating counts as measures of relative rather than absolute abundance.

In conclusion, we urge researchers to evaluate and recognize that a few mobile predator species, most clearly *A*. *virescens* and *C*. *melampygus*, have been shown to be attracted to OC bubbles in remote locations, arriving earlier in the surveys and in greater numbers. These tendencies will influence surveys, and the extent of influence will depend to a large degree on method. For example, bias caused by OC would be much greater for short counts conducted soon after a diver arrived at the location. Additionally, these results may vary based on survey method. The strip transect is a more active survey approach that requires the diver to constantly move forward and may result with an increased flight response. However, whatever the method, there certainly is potential for attraction of roving piscivores to divers, particularly if using OC SCUBA, within highly protected remote areas, thus artificially increasing counts of those species, and thereby inflating apparent differences with areas in which those species have learned to be cautious of divers.

## Supporting information

S1 Table Site coordinates. (PDF)

S2 Table Table of fish abundance and biomass between gear types. (PDF)

S3 Table Number of observed fishes for both gear types. (PDF)

S4 Table Chi-squared test of distribution results for the instance of first encounter. (PDF)

S1 Fig Abundance and biomass ratio for all species observed in at least 15 surveys. (PDF)

S1 Dataset Raw data from the 35 paired fish surveys conducted in 2017. (XLSX)
