# Supplementary material for: Effects of SCUBA bubbles on counts of roving piscivores in a large remote marine protected area
Source: PLoS One. 2019 Dec 18;14(12):e0226370. doi: 10.1371/journal.pone.0226370 (PMC6919603; doi:10.1371/journal.pone.0226370)
Supplement: S1 Table — (PDF) [file pone.0226370.s001.pdf]

S1 Table: Site Coordinates. Site coordinates by atoll. Lalo (French Frigate Shoals), Kamole (Laysan), Kapou (Lisianski), Manawai (Pearl and Hermes), Kuaihelani (Midway), Hōlanikū (Kure).

Atoll	Depth (m)	Latitude	Longitude
Lalo	14.1	23.8809 N	-166.2249
(French Frigate Shoals)	25.8	23.8834 N	-166.2495
	17.0	23.8800 N	-166.2775
	10.0	23.8593 N	-166.3224
	24.8	23.8313 N	-166.3202
	20.4	23.6354 N	-166.1855
	15.2	23.8467 N	-166.1446
	16.7	23.8605 N	-166.1673
	10.0	23.6548 N	-166.2047
Kamole	25.0	25.8016 N	-171.7114
(Laysan)	22.3	25.7467 N	-171.7509
	15.5	25.7879 N	-171.7357
	17.0	25.7752 N	-171.7556
	14.3	25.7765 N	-171.7501
	17.6	25.7817 N	-171.7043
Kapou	13.0	26.0394 N	-174.0138
(Lisianski)	19.7	25.9162 N	-173.9518
	13.0	25.9506 N	-173.9666
	13.1	26.0825 N	-173.9994
	24.2	26.0962 N	-173.9508
	16.1	26.0610 N	-174.0173
	24.5	26.0790 N	-173.9089
	12.4	26.0568 N	-173.9113
	23.2	26.0669 N	-173.8816

Manawai	22.4	27.7590 N	-175.9703
(Pearl and Hermes)	17.0	27.7596 N	-175.9282
	23.6	27.8568 N	-175.7337
	19.1	27.9009 N	-175.7210
	22.8	27.8338 N	-175.7416
Kuaihelani	22.2	28.1935 N	-177.3493
(Midway)	20.4	28.1939 N	-177.3788
Hōlanikū	22.1	28.3806 N	-178.3250
(Kure)	12.4	28.4023 N	-178.2778
	13.3	28.3853 N	-178.3481
	16.4	28.3817 N	-178.3000
