# Supplementary material for: Effects of SCUBA bubbles on counts of roving piscivores in a large remote marine protected area
Source: PLoS One. 2019 Dec 18;14(12):e0226370. doi: 10.1371/journal.pone.0226370 (PMC6919603; doi:10.1371/journal.pone.0226370)
Supplement: S2 Table — (PDF) [file pone.0226370.s002.pdf]

S2 Table: Table of fish abundance and biomass between gear types. Table of analysis including the mean and standard error of density (abundance or biomass) for OC and CCR surveys. Abundance is measured in fish per hectare and biomass is measured in grams per m^2. The abundance (or biomass) ratio is the ratio between OC and CCR densities, here represented as mean ratio (AR or BR) and 95% low and high confidence intervals of that ratio. Differences between OC and CCR are considered significant when the 95% confidence intervals of the AR or BR do not overlap 1. There were 35 surveys conducted on each gear type however not all species were observed in all transects.

Group	OC			CCR			OC:CCR Ratio		Results
	N	Mean	SE	N	Mean	SE	Ratio	CI (95%)	On OC
All Fish Biomass (g/m²)	35	226.8	40.1	35	176.0	41.6	1.29	(0.85,1.57)	n.s.
Species Richness (no./survey)	35	38.4	1.7	35	39.7	1. 7	0.97	(0.93, 1.01)	n.s.
All Fish Abundance (no./Ha)	35	2,494.7	240.7	35	2,706.5	296.1	0.92	(0.66, 1.06)	n.s.
Abundance Ratios (no./Ha)									
Trevally Jacks	28	166.5	60.4	23	108.3	37.0	1.54	(1.06, 2.43)	~50% more
Benthic Damselfish/Angelfish	32	1,728.4	527.6	32	1,261.9	307.3	1.37	(1.06, 1.95)	~40% more
Butterflyfish	35	343.6	47.9	35	437.3	75.1	0.79	(0.40, 0.95)	~20% fewer
Goatfish	15	31.5	13.9	12	28.3	10.7	1.11	(0.66, 1.63)	n.s.
Hawkfish	18	143.1	51.6	21	152.0	49.4	0.94	(0.52, 1.18)	n.s.
Midwater Damselfish	30	1,701.7	479.0	29	1,324.2	421.2	1.29	(0.54, 2.08)	n.s.
Parrotfish	26	92.2	16.3	25	105.1	19.0	0.88	(0.68, 1.09)	n.s.
Surgeonfish	30	139.0	35.3	33	145.5	22.7	0.96	(0.66, 1.41)	n.s.
Sharks	21	43.7	16.1	15	33.1	8.1	1.32	(0.78, 2.77)	n.s.
Triggerfish	26	67.9	9.3	26	80.0	15.5	0.85	(0.47, 1.07)	n.s.
Targeted wrasse	34	62.2	4.3	33	63.1	5.8	0.99	(0.74, 1.21)	n.s.
Non-targeted wrasse	35	852.1	71.2	35	854.5	62.4	1.00	(0.88, 1.15)	n.s.
Caranx melampygus	17	51.7	17.1	17	24.3	5.5	2.13	(1.30, 4.00)	~2x as many
Caranx ignobilis	20	102.7	47.5	13	65.5	33.6	1.57	(1.07, 2.47)	~60% more
Aprion virescens	24	42.0	6.3	25	41.2	6.0	0.99	(0.74, 1.21)	n.s.
Carcharhinus galapagensis	10	27.5	16.2	9	21.8	7.2	1.26	(0.56, 3.63)	n.s.
Trianodon obesus	10	13.7	4.1	7	9.7	3.7	1.42	(0.50, 2.58)	n.s.
Biomass Ratios (g/m2)									
Trevally Jacks	28	124.1	35.8	23	79.3	29.5	1.57	(1.08, 2.12)	~60% more
Butterflyfish	35	1.2	0.2	35	1.6	0.2	0.75	(0.54, 0.94)	~25% less
Benthic Damselfish/Angelfish	32	0.6	0.1	32	0.5	0.1	1.21	(0.96, 1.72)	n.s.
Hawkfish	18	0.2	0.1	21	0.2	0.1	1.14	(0.72, 1.73)	n.s.
Midwater Damselfish	30	1.6	0.4	29	2.4	0.8	0.69	(0, 1.15)	n.s.
Parrotfish	26	4.7	0.9	25	4.5	0.9	1.05	(0.78, 1.37)	n.s.
Surgeonfish	30	8.6	4.9	33	5.3	1.3	1.63	(0.83, 4.63)	n.s.
Sharks	21	51.4	19.4	15	51.2	19.2	1.00	(0.21, 1.91)	n.s.
Triggerfish	26	1.3	0.3	26	1.3	0.3	1.00	(0.71, 1.29)	n.s.
Targeted wrasse	29	2.6	0.5	30	1.9	0.4	1.38	(0.9, 2.01)	n.s.
Non-targeted wrasse	35	2.6	0.3	35	2.4	0.3	1.11	(0.94, 1.36)	n.s.
Caranx melampygus	17	14.9	5.7	17	6.0	1.9	2.48	(1.37, 4.74)	~2.5x as many
Caranx ignobilis	20	107.6	32.8	13	68.4	28.3	1.57	(1.1, 2.17)	~60% more
Aprion virescens	24	15.3	2.9	25	14.3	2.6	1.07	(0.72, 1.42)	n.s.
Carcharhinus galapagensis	10	37.2	19.7	9	43.6	19.2	0.85	(0, 1.9)	n.s.
Trianodon obesus	10	10.4	3.3	7	5.3	2.1	1.96	(0.88, 3.27)	n.s.
