# Supplementary material for: Effects of SCUBA bubbles on counts of roving piscivores in a large remote marine protected area
Source: PLoS One. 2019 Dec 18;14(12):e0226370. doi: 10.1371/journal.pone.0226370 (PMC6919603; doi:10.1371/journal.pone.0226370)
Supplement: S3 Table — (PDF) [file pone.0226370.s003.pdf]

S3 Table: Number of observed fishes for both gear types. List of fish species which had no significant differences between gear types. This table shows the number of individual fishes counted by divers using the respective gear types.

Taxon		OC	CCR
Acanthuridae	*Acanthurus blochii*	14	6
	Acanthurus dussumieri	2	15
	Acanthurus leucopareius	12	10
	Acanthurus nigroris	189	114
	Acanthurus olivaceus	129	115
	Acanthurus triostegus	210	199
	Ctenochaetus strigosus	370	394
	Naso hexacanthus	51	124
	Naso lituratus	*19*	*23*
	Naso unicornis	73	68
	Zebrasoma flavescens	60	48
Carangidae	*Decapterus macarellus*	300	160
Labridae	*Bodianus albotaeniatus*	77	78
Lethrindae	*Monotaxis grandoculis*	37	55
Lutjanidae	*Aphareus furca*	18	13
	Aprion virescens	52	51
	Lutjanus kasmira	108	37
Mullidae	*Parupaneus cyclostomus*	36	38
	Parupeneus multifasciatus	126	117
	Parupeneus porphyreus	12	13

Scaridae	*Calotomus carolinus*	22	25
	Calotomus zonarchus	12	7
	Chlorurus perspicillatus	85	83
	Chlorurus spilurus	118	108
	Scarus psittacus	14	14
	Scarus rubroviolaceus	3	0
	All Species	2406	2090
