# Supplementary material for: Effects of SCUBA bubbles on counts of roving piscivores in a large remote marine protected area
Source: PLoS One. 2019 Dec 18;14(12):e0226370. doi: 10.1371/journal.pone.0226370 (PMC6919603; doi:10.1371/journal.pone.0226370)
Supplement: S4 Table — (PDF) [file pone.0226370.s004.pdf]

S4 Table: **Chi-squared test of distribution results for the instance of first encounter.** The results for the Chi-squared test of distributions for the 5 species of interest and the associated Chi-squared numbers and its p-value.

Species	Chi-squared Number	p-value
Aprion virescens	6.6114	0.04*
Carcharhinus galapagensis	1.6908	0.43
Triaenodon obesus	7.6912	0.02*
Caranx ignobilis	1.0014	0.61
Caranx melampygus	22.021	<0.001*

* Significant Difference
