# Supplementary material for: Effects of SCUBA bubbles on counts of roving piscivores in a large remote marine protected area
Source: PLoS One. 2019 Dec 18;14(12):e0226370. doi: 10.1371/journal.pone.0226370 (PMC6919603; doi:10.1371/journal.pone.0226370)
Supplement: S1 Fig — (PDF) [file pone.0226370.s005.pdf]

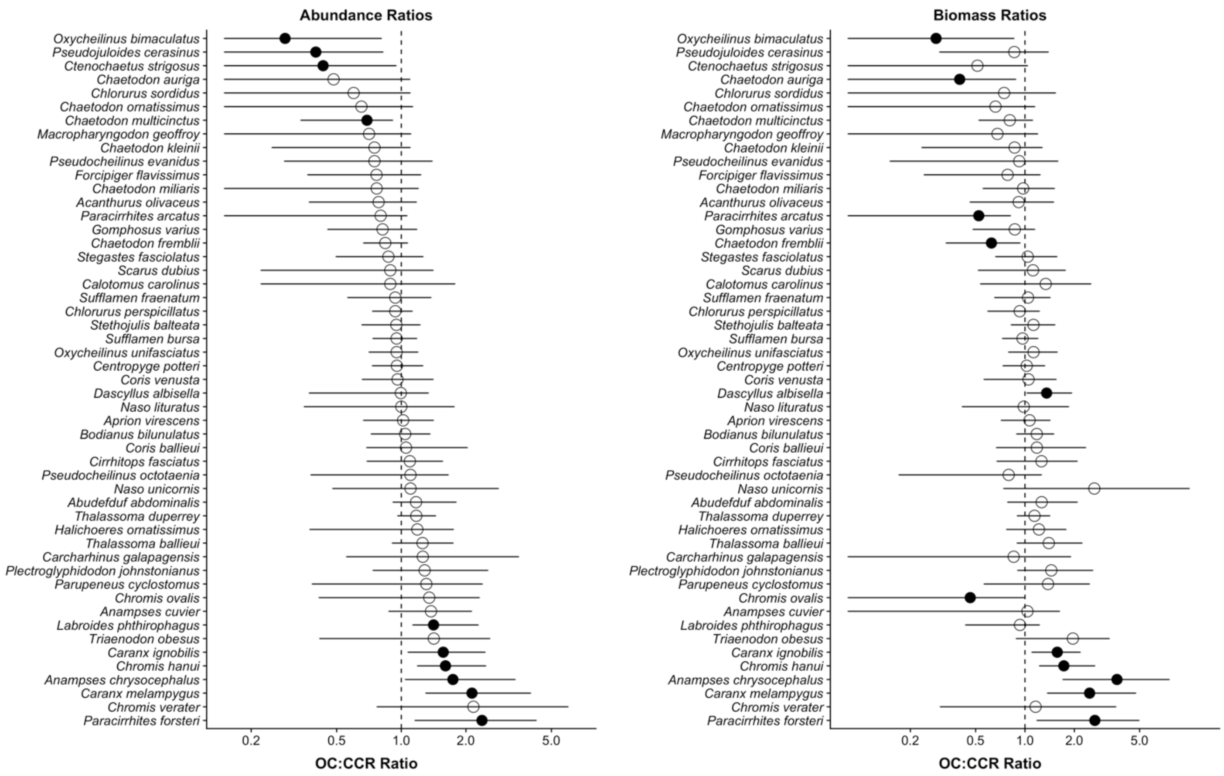

S1 Figure: **Abundance and biomass ratio for all species observed in at least 15 surveys** (n total of 70 surveys conducted = 35 on OC, and 35 on CCR). Species are sorted by OC:CCR abundance ratio (i.e. high values represent cases where more fishes counted in OC than CCR). Abundance and biomass ratio low values limited to 1:12 for biomass and 1:6 for abundance.
